# Effect of homeostatic T-cell proliferation in the vaccine responsiveness against influenza in elderly people

**DOI:** 10.1186/s12979-019-0154-y

**Published:** 2019-07-05

**Authors:** I. Herrero-Fernández, I. Rosado-Sánchez, A. I. Álvarez-Ríos, M. I. Galvá, M. De Luna-Romero, S. Sanbonmatsu-Gámez, M. Pérez-Ruiz, J. M. Navarro-Marí, A. Carrillo-Vico, B. Sánchez, R. Ramos, J. Cañizares, M. Leal, Y. M. Pacheco

**Affiliations:** 1 Institute of Biomedicine of Seville (IBiS), Virgen del Rocío University Hospital (HUVR)/CSIC/University of Seville, Seville, Spain; 2 Department of Clinical Biochemistry, Virgen del Rocío University Hospital, Seville, Spain; 3 Heliopolis Nursing Home, Seville, Spain; 4 Servicio de Microbiología, Hospital Universitario Virgen de las Nieves, Instituto de Investigación Biosanitaria Ibs Granada, Granada, Spain; 5 Department of Medical Biochemistry, Molecular Biology and Immunology, University of Seville, Seville, Spain; 6 Immunology Service, Instituto de Biomedicina de Sevilla, Hospital Universitario Virgen del Rocío/CSIC/Universidad de Sevilla, Seville, Spain; 7 Immunovirology section, Viamed Hospital, Santa Ángela de la Cruz, Seville, Spain; 8 Laboratory of Immunology, Institute of Biomedicine of Seville (IBiS)/UGC Clinical, Laboratories, Virgen del Rocío University Hospital, Avda. Manuel Siurot s/n. PC, 41013 Seville, Spain

**Keywords:** Treg, Ki67, Inflammation, Thymic function, TREC

## Abstract

**Background:**

Seasonal influenza virus infection is a significant cause of morbimortality in the elderly. However, there is poor vaccine efficacy in this population due to immunosenescence. We aimed to explore several homeostatic parameters in the elderly that could impact influenza vaccine responsiveness.

**Methods:**

Subjects (> 60 years old) who were vaccinated against influenza virus were included, and the vaccine response was measured by a haemagglutination inhibition (HAI) test. At baseline, peripheral CD4 and CD8 T-cells were phenotypically characterized. Thymic function and the levels of different inflammation-related biomarkers, including Lipopolysaccharide Binding Protein (LBP) and anti-cytomegalovirus (CMV) IgG antibodies, were also measured.

**Results:**

Influenza vaccine non-responders showed a tendency of higher frequency of regulatory T-cells (Tregs) before vaccination than responders (1.49 [1.08–1.85] vs. 1.12 [0.94–1.63], respectively, *p* = 0.061), as well as higher expression of the proliferation marker Ki67 in Tregs and different CD4 and CD8 T-cell maturational subsets. The levels of inflammation-related biomarkers correlated with the frequencies of different proliferating T-cell subsets and with thymic function (e.g., thymic function with D-dimers, r = − 0.442, *p* = 0.001).

**Conclusions:**

Age-related homeostatic dysregulation involving the proliferation of CD4 and CD8 T-cell subsets, including Tregs, was related to a limited responsiveness to influenza vaccination and a higher inflammatory status in a cohort of elderly people.

**Electronic supplementary material:**

The online version of this article (10.1186/s12979-019-0154-y) contains supplementary material, which is available to authorized users.

## Background

The seasonal influenza virus (flu) is a significant cause of morbidity and mortality in older adults [1]. The World Health Organization (WHO) estimates that 3–5 million cases of severe influenza illness and up to 650,000 deaths related to respiratory diseases are linked to seasonal flu each year [2], with the highest mortality rates occurring in the elderly [3]. Despite vaccination remaining the most effective approach for the prevention of influenza infection and influenza-related complications, there is poor vaccine efficacy in the elderly [4], which is due to the age-associated dysregulation of immune function known as immunosenescence [5, 6].

Immunosenescence affects both the innate and adaptive branches of the immune system. Thus, age-related alterations, such as those affecting Toll-like receptors [7], reduced telomerase activity [8] and deficiencies in B and T-cell functions [9, 10], have been associated with influenza vaccine responsiveness. Moreover, elderly people exhibit a chronic inflammatory status, called inflammaging, with increased levels of circulating inflammatory mediators such as pro-inflammatory cytokines and acute phase proteins, e.g., interleukin-6 (IL-6) and C-reactive protein (CRP), respectively, that disturb vaccine responses [11], specifically, the influenza vaccine response [12, 13]. Several factors, mainly persistent stressors such as translocated microbial products (LPS) and cytomegalovirus coinfection (CMV) but also the age-related increasing activation of the coagulation system (D-dimers), have been proposed to contribute to this age-related inflammation [14]. Interestingly, regarding the adaptive immune system, naïve T-cells retain their proliferative capacity in both aged mice and humans [15] and naïve T-cell proliferation can even be enhanced in the elderly because of the age-dependent loss of thymic output [16–18]. This immunosenescence-related homeostatic dysregulation (mainly the thymic output – compensatory peripheral T-cell proliferation axis) could also be related to inflammaging and even affect immune competence in the elderly. However, the potential role of this homeostatic dysregulation in vaccine responsiveness is mostly unexplored.

The proliferation and cytokine secretion of CD4 and CD8 T-cells is regulated by regulatory T-cells (Tregs), which are also involved in the suppression of antigen presenting cells (APC) and B cells [19]. In fact, Tregs suppress the B cell immunoglobulin class switching within germinal centres of human lymphoid tissue [20]. Because of that observation, Tregs have been explored in several immunization models in animals [21, 22] and in humans [23, 24]. Particularly, in the context of influenza vaccination, despite Tregs being known to expand after vaccination, which possibly attenuates the production of anti-influenza antibodies [25, 26], the role of baseline Tregs remains mostly unexplored. However, this is an interesting question since Treg frequency increases with age [27], probably as another consequence of age-dependent homeostatic dysregulation [28, 29].

In the present work, we aimed to further explore the potential association between several immune homeostatic parameters in the elderly, such as thymic function, T-cell proliferation, Tregs and several inflammation- and coagulation-related markers, and influenza vaccine responsiveness.

## Results

### Rates of vaccine response

Sixty subjects were included in this study. The demographic and clinical characteristics of these subjects are summarized in Table 1. Briefly, 24/60 (40%) of the subjects were men, and the median age was 79 [70–87] years. Before vaccination, the subjects showed a median CD4/CD8 T-cell ratio of 1.8 [1.2–2.3] and a broad range of thymic function measured as an sj/β-TREC ratio of 32 [0–50]. Seroprotection was present in 48/60 (80%) of the subjects before vaccination and in 59/60 (98%) of the subjects after vaccination. A vaccine response was observed in 27/60 (45%) of the subjects. The baseline and post-vaccination HAI titres for the whole group, as well as the titres for the responder and non-responder groups, are shown in Additional file 6: Figure S1. The timing of the post-vaccination sampling did not affect the HAI titres or the seroconversion-fold data (data not shown). No differences were observed in the number of comorbidities or the disability degree between the studied groups.

**Table 1:** Characterization of the study population. Comparisons between groups regarding the vaccine response to the influenza vaccine

Variable	TOTAL *N* = 60	Non-Responders *N* = 33	Responders N = 27	*p*
**Age (years)**	79 [70–87]	80 [67–88]	77 [71–86]	0.806
**Male sex, n (%)**	24 (40)	15 (46)	9 (33)	0.340
**CD4** ^**+**^ **T-cell count** **(cells/mm** ^**3**^ **)**	799 [614–1103]	715 [533–1236]	825 [749–1008]	0.281
**CD8** ^**+**^ **T-cell count** **(cells/mm** ^**3**^ **)**	473 [279–685]	439 [159–671]	486 [384–698]	0.185
**CD4** ^**+**^ **/CD8** ^**+**^ **ratio**	1.8 [1.2–2.3]	1.9 [1.2–2.3]	1.6 [1.1–2.7]	0.704
**sj/β TREC ratio**	32 [0–50]	31 [0–55]	34 [7–50]	0.615
**Thymic failure** ^a^	19 (32)	12 (36)	7 (27)	0.441
**CMV titre (AU/μL)**	25.4 [13.3–40.3]	25.4 [12.4–39.0]	24.6 [13.8–43.0]	0.973
**LBP (ng/μL)**	12.7 [10.2–14.1]	13.0 [10.1–15.3]	12.7 [11.4–13.5]	0.564
**hsCRP (mg/L)**	2.8 [1.6–5.0]	2.3 [1.2–4.8]	3.1 [2.2–5.0]	0.330
**B2M (μg/mL)**	2.5 [2.1–3.5]	2.7 [2.4–3.7]	2.3 [2.0–3.3]	0.156
**D-dimers (μg/L)**	705 [438–1183]	875 [445–1425]	620 [438–918]	*0.082*
**IL-6 (pg/mL)**	3.5 [2.4–4.6]	3.4 [2.3–4.9]	3.6 [2.6–4.5]	0.691
**sCD163 (ng/L)**	1034 [844–1293]	1034 [792–1224]	1084 [878–1477]	0.222
**% Lymphocytes**	26.2 [23.2–33.1]	24.9 [22.4–32.0]	29.0 [25.0–34.1]	*0.063*
**% Monocytes**	6.5 [5.4–7.6]	6.6 [5.4–7.9]	6.5 [5.4–7.5]	0.894
**% Neutrophils**	59.6 [54.1–65.7]	62.8 [54.4–66.9]	58.0 [52.4–62.5]	*0.089*
**% Basophils**	0.2 [0.2–0.4]	0.3 [0.2–0.4]	0.2 [0.1–0.3]	0.258
**% Eosinophils**	3.4 [2.2–4.2]	3.2 [1.7–4.5]	3.7 [3.2–4.2]	*0.094*
**Platelets (x10e9/L)**	223 [180–294]	233 [181–294]	197 [170–296]	0.650
**MCV (fL)**	90.2 [86.1–93.6]	90.6 [85.5–93.5]	90.0 [86.6–95.1]	0.876
**MPV (fL)**	9.4 [7.9–10.1]	9.3 [7.8–10.3]	9.40 [8.1–10.0]	0.716
**ESR (mm/h)**	12 [6–22]	14 [6–21]	10 [6–24]	0.921
**PLR**	117 [87–164]	134 [88–166]	113 [82–144]	0.377
**NLR**	2.3 [1.6–2.9]	2.6 [1.7–3.0]	1.9 [1.5–2.4]	*0.056*
**Comorbidities (number** ^b^ **)**	3 [2–5]	3 [2–5]	3 [2–5]	0.874
**Barthel index** ^c^	87 [70–100]	85 [70–100]	90 [70–100]	0.838
**< 20**	3 (5)	1 (3.8)	2 (6.1)	
**20–35**	2 (3.3)	1 (3.8)	1 (3)	
**40–55**	5 (8.3)	3 (11.5)	2 (6.1)	
**≥ 60**	32 (53.3)	14 (53.8)	18 (54.5)	
**100**	18 (30)	8 (29.6)	10 (30.3)	

Continuous variables are expressed as median values [IQR], and categorical variables are expressed as the number of cases (%). Comparisons between the groups were made using the nonparametric Mann–Whitney *U* test for continuous variables and the χ2 or Fisher exact test for categorical variables. Variables with a *p* value < 0.1 are shown in *italics*. Variables with a *p* value < 0.05 were considered statistically significant and are shown in bold. Note: *CMV* Cytomegalovirus, *LBP* Lipopolysaccharide Binding Protein, *hsCRP* High sensitivity C-Reactive Protein, *B2M* β2-microglobulin, *sCD163* soluble CD163, *MCV* Mean corpuscular volume, *MPV* Mean platelet volume, *ESR* erythrocyte sedimentation rate, *PLR* Platelet to lymphocyte ratio, and *NLR* Neutrophil to lymphocyte ratio. ^(a)^ Thymic failure is defined as an sj/β TREC ratio < 10. ^b^Details of comorbidities recorded are shown in Additional file 5: Table S5. ^c^100 is totally independent and < 20 is totally dependent

### Levels of inflammation-related markers according to vaccine responsiveness

The non-responders did not differ from the responders in terms of age, sex, CD4/CD8 ratio, sj/β-TREC ratio or anti-cytomegalovirus (CMV) titre (Table 1). Interestingly, several nonsignificant differences in inflammation-related markers were observed. Specifically, the non-responders showed higher levels of D-dimers (875 [445–1425] vs 620 [438–918], respectively; *p* = 0.082), higher % neutrophils (62.8 [54.3–66.9] vs 58.0 [52.4–62.5], respectively; *p* = 0.089) and higher neutrophil to lymphocyte ratio (NLR) (2.6 [1.7–3.0] vs 1.9 [1.5–2.4], respectively; *p* = 0.056) than the responders, though without statistical significance. The non-responders showed lower % lymphocytes (24.9 [22.4–31.9] vs 29.0 [25.0–34.1], respectively; *p* = 0.063) and % eosinophils (3.15 [1.73–4.45] vs 3.70 [3.20–4.20], respectively; *p* = 0.094), but also without statistical significance.

### Frequency of Tregs and Tregs expressing Ki67 according to vaccine responsiveness

**Fig. 1:**
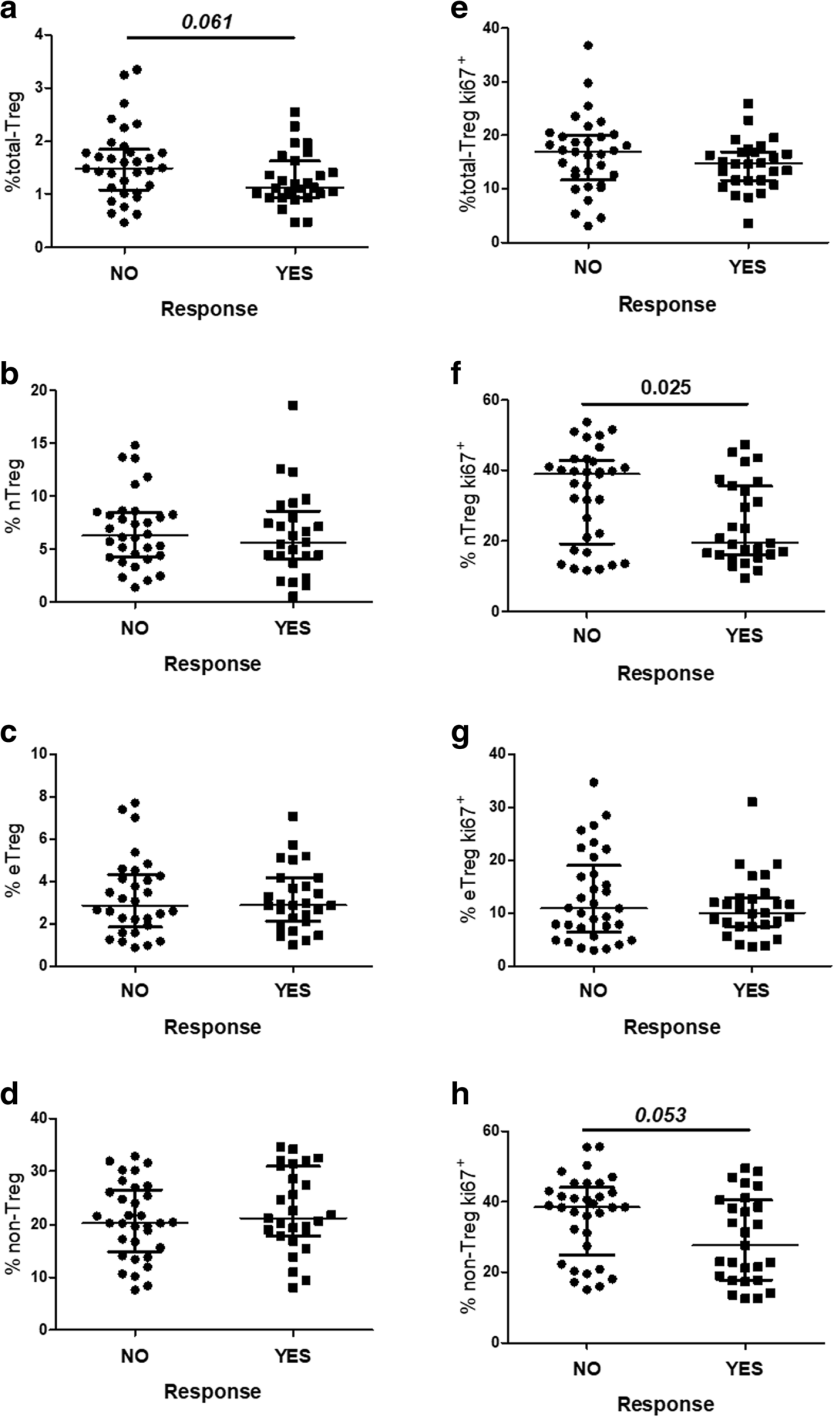
Characterization of Treg subsets in relation to the response to the influenza vaccine. **a-d** Frequencies of Treg subsets. **e-h** Frequencies of Treg subsets expressing the proliferation marker Ki67^+^. A total of 60 subjects, within an age range of 61–98 years old (min-max) and median age of 79 [70–87] (median [IQR]) were included. Comparisons between the groups of vaccine non-responders (n = 33) and responders (n = 27) were made using the nonparametric Mann–Whitney *U* test. Variables with a *p* value < 0.05 were considered statistically significant and are shown in bold. *After the Bonferroni correction for multiple comparisons, the comparison of % nTregKi67+ did not remain statistically significant. Note: nTreg, naïve-Treg; and eTreg, effector-Treg

We explored the frequencies of total-Tregs and Treg subsets and the expression of different activation, proliferation and suppression markers in relation to influenza vaccine responsiveness (Fig. 1 and Additional file 1: Table S1). We observed a tendency of higher frequency of total-Tregs in the non-responders compared with the responders; however, the difference was not significant (1.49 [1.08–1.85] vs 1.12 [0.94–1.63], respectively; *p* = 0.061). However, when the two groups were split according to the total-Treg frequency by using the overall median value (1.38) as a cutoff, 22/33 (67%) of the non-responders but only 8/27 (30%) of the responders showed a Treg frequency above the median (*p* = 0.004). No differences were observed regarding the frequencies of Treg subsets. Nevertheless, the non-responders presented higher frequencies of both naïve-Tregs (nTregs) (38.9 [19.2–42.8] vs 19.5 [16.1–35.6], respectively; *p* = 0.025) and non-Tregs (38.5 [24.9–44.1] vs 27.7 [17.9–40.4], respectively; *p* = 0.053), expressing the proliferation marker Ki67; however, statistical significance was reached with only the nTregs.

### Frequencies of CD4 and CD8 T-cell maturational subsets expressing Ki67 according to vaccine responsiveness

**Fig. 2:**
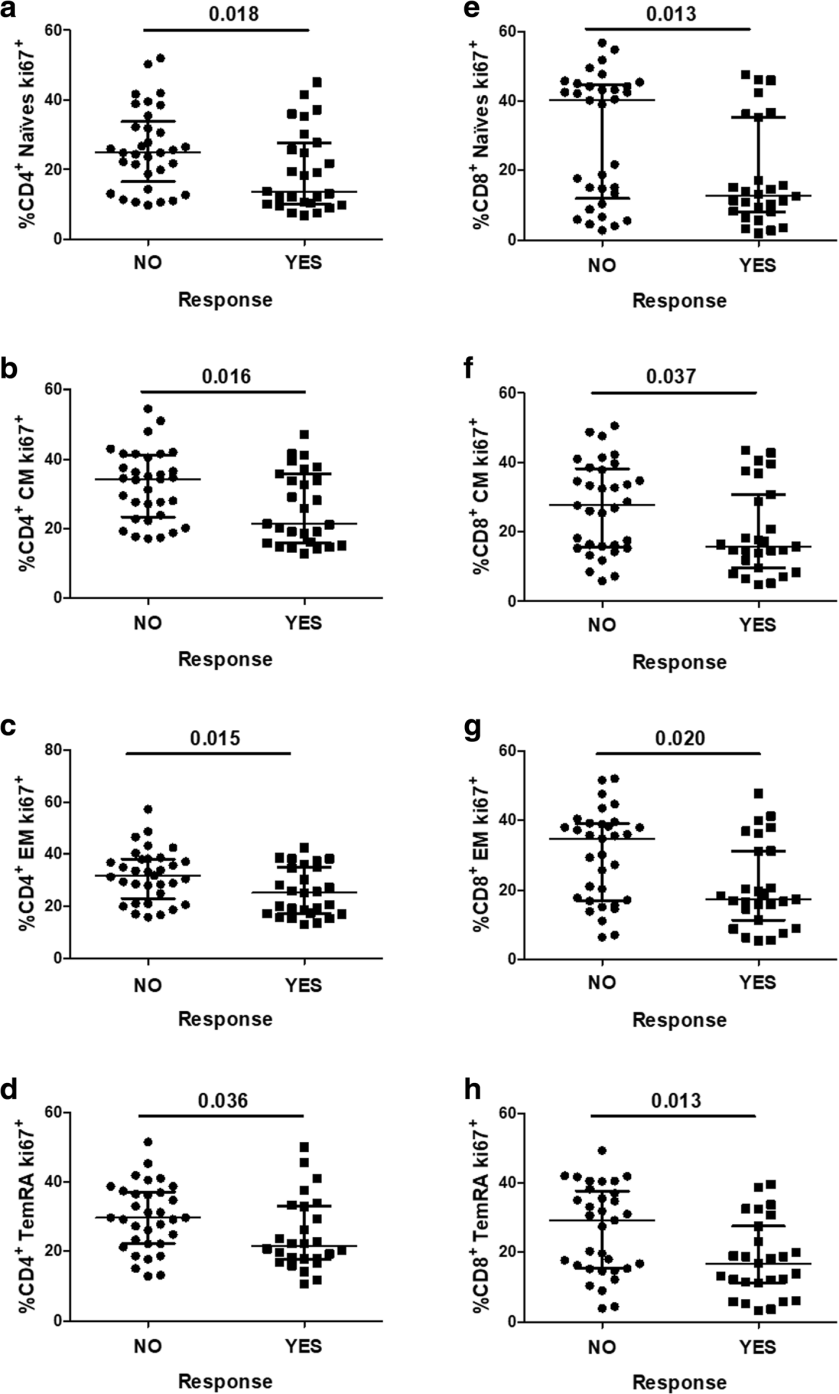
Maturational subsets of CD4^+^ and CD8^+^ T-cells expressing Ki67^+^. **a**-**d** Frequencies of CD4^+^ maturational subsets expressing the proliferation marker Ki67^+^. **e-h** Frequencies of CD8^+^ maturational subsets expressing the proliferation marker Ki67^+^. Comparisons between the groups of vaccine non-responders (*n* = 33) and responders (*n* = 27) were made using the nonparametric Mann–Whitney *U* test. Variables with a *p* value < 0.05 were considered statistically significant and are shown in bold. *After correction for multiple comparisons by the Benjamini-Hochberg procedure, applying a 10% FDR, all statistical significances remained. Note: CM, central memory; EM, effector memory; and TemRA, terminally differentiated effector memory

We examined CD4 and CD8 T-cell maturational subsets and the expression of different markers of activation (HLA-DR), apoptosis susceptibility (CD95), senescence (CD57), proliferation (Ki67) and suppression (CTLA-4) in CD4 and CD8 T-cell pools. We found no differences between the vaccine responsiveness groups in either, the distribution of the maturational subsets or the expression of the abovementioned cellular markers (Additional file 2: Table S2). We also analysed the expression of Ki67 specifically on the different CD4 and CD8 maturational subsets to study the proliferation of these subsets. We observed higher expression of Ki67 in both the CD4 and CD8 maturational subsets from the non-responders compared to those from the responders, and these differences in expression reached statistical significance for all comparisons (Fig. 2).

### Associations among inflammation-related biomarkers, the expression of Ki67 in T-cells and thymic function

**Fig. 3:**
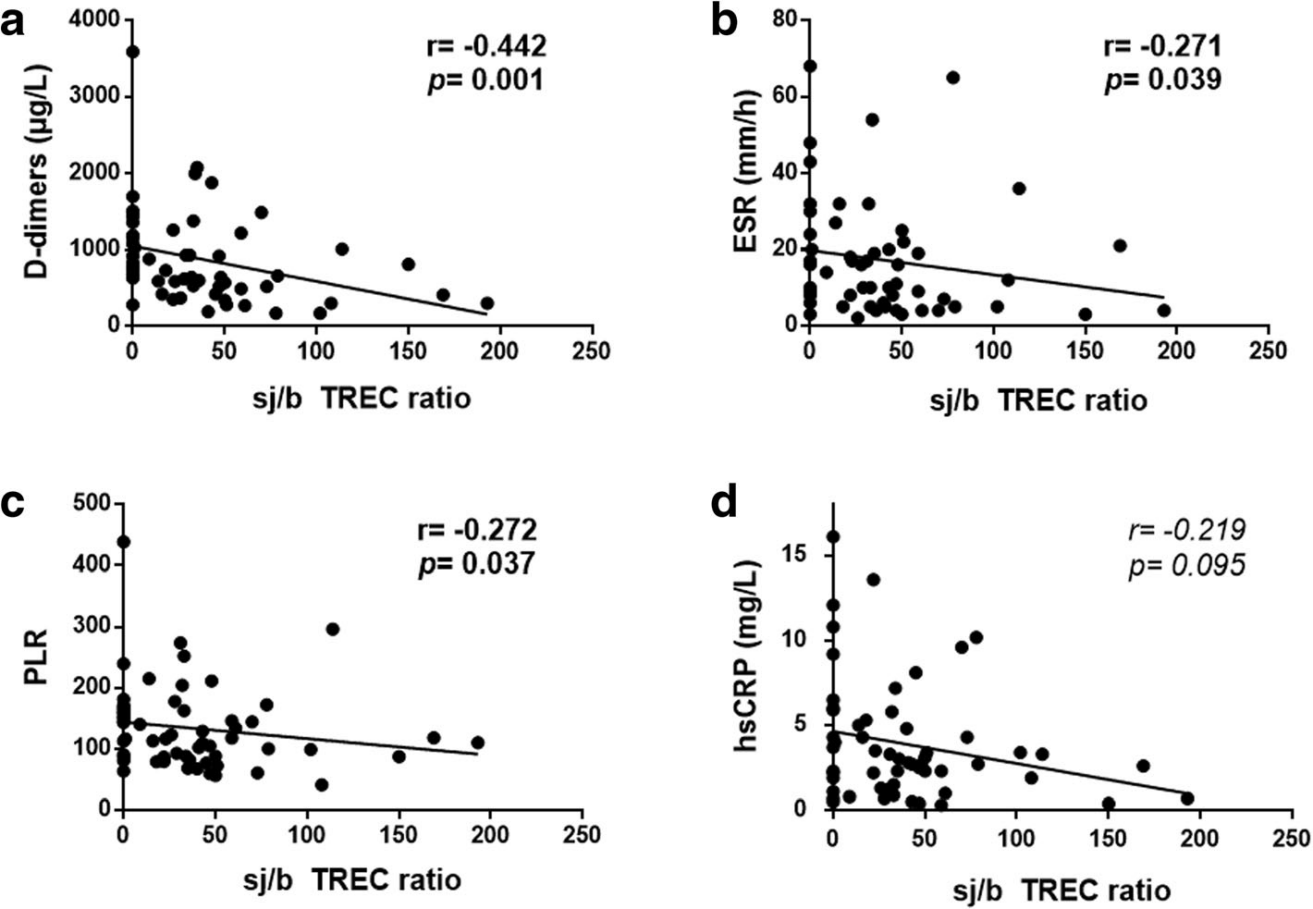
Correlations between the sj/β TREC ratio and inflammation-related biomarkers. **a** Correlation between sj/β TREC and D-dimers. **b** Correlation between sj/β TREC and the ESR. **c)** Correlation between sj/β TREC and the PLR. **d** Correlation between sj/β TREC and hsCRP. A total of 60 subjects. Correlations were assessed using Spearman’s rho correlation coefficient. Variables with a *p* value < 0.1 are shown in *italics*. Variables with a *p* value < 0.05 were considered statistically significant and are shown in bold. *After correction for multiple comparisons by the Benjamini-Hochberg procedure, applying a 10% FDR, all statistical significances remained. Note: ESR, erythrocyte sedimentation rate; PLR, platelets to lymphocyte ratio; and hsCRP, high sensitivity C-Reactive Protein

We explored the potential associations of the analysed inflammation-related biomarkers and haematological parameters with the expression of Ki67 in different Treg subsets (naïve Tregs –nTregs– and effector Tregs –eTregs–) as well as in different CD4 and CD8 T-cell maturational subsets. Interestingly, we observed positive associations between Ki67 expression in several T-cell subsets and Lipopolysaccharide Binding Protein (LBP) levels, high sensitivity C reactive protein (hsCRP) levels (e.g., with %eTreg-Ki67^+^; r = 0.301, *p* = 0.020), β2-microglobulin levels (e.g., with %CD4^+^Ki67^+^; r = 0.322, *p* = 0.010), D-dimer levels (e.g., with %CD4^+^Ki67^+^; r = 0.446, *p* = 0.001), the % monocytes, the % neutrophils (e.g., with %CD4^+^EM Ki67^+^; r = 0.307, *p* = 0.017), the % basophils, the platelet to lymphocyte ratio (PLR) and the NLR, while there were negative associations between the Ki67 expression in several T-cell subsets and the % lymphocytes (e.g., with %CD4^+^EM Ki67^+^; r = − 0.310, *p* = 0.016), % eosinophils, Mean Corpuscular Volume (MCV) (e.g., with CD8 + TemRA Ki67+; r = − 0.303, *p* = 0.019) and Mean Platelet Volume (MPV) (e.g., with %nTreg Ki67+; r = − 0.550, *p* < 0.001) (Additional file 3: Table S3). We also observed associations between thymic function and the levels of inflammation-related biomarkers (D-dimers, Erythrocyte Sedimentation Rate (ESR) and the PLR) as well as a trend with hsCRP levels (Fig. 3). Moreover, two of the inflammation-related biomarkers (the ESR and LBP levels) also tended to correlated with the anti-CMV titre (r = 0.313, *p* = 0.021 and r = 0.263, *p* = 0.057; respectively). Nevertheless, as indicated in the Table footnote, not all these associations remained statistically significant after the Bonferroni correction for multiple comparisons.

Remarkably, we found higher levels of inflammation-related biomarkers and expression of Ki67^+^ in T-cell subsets and lower thymic function in the subjects who died during a year-long follow-up (Additional file 4: Table S4). Six subjects (one responder and five non-responders) died during this follow-up period as a consequence of cardiovascular events, and despite acknowledging the low number of events, we explored the baseline immune characteristics of these subjects. As expected, the subjects who died showed higher baseline levels of hsCRP than those who survived (5.10 [3.70–7.58] vs 2.60 [1.25–4.30], respectively; *p* = 0.012). However, interestingly, we also found higher frequencies of T-cell subsets, including Tregs, expressing Ki67 in the subjects who died. Furthermore, the six subjects who died during the follow-up year had lower thymic function (0 [0–0] vs. 34 [10–51], respectively; *p* = 0.001), and all of these subjects showed a failure in thymic function. Subjects who died during the follow-up did not show a statistically higher number of comorbidities, but they had a higher disability degree.

## Discussion

We report that the influenza vaccine responsiveness of an aged population was associated with age-related homeostatic dysregulation involving T-cell proliferation in CD4 and CD8 T-cell maturational subsets. Moreover, higher frequencies of not only CD4 Tregs but also proliferating Treg subsets were associated with vaccine non-response. Overall, this homeostatic dysregulation was directly correlated with the inflammatory status in this context.

Tregs are involved in the suppression of the immune system and prevent a proper antibody response to vaccination [19, 20]. Herein, we studied the frequencies and several functional markers of Treg subsets in relation to the influenza vaccine response of elderly people. Interestingly, we observed a tendency of higher baseline frequency of total-Tregs in non-responders than responders, a comparison that became statistically significant when considering the number of subjects in each group showing a higher than the median Treg frequency. Our results are in line with those of van de Geest et al. [30], although those authors specifically found higher frequencies of the effector-Treg subset. As far as we know, no other previous studies focusing on baseline Tregs in the elderly in relation to vaccine responsiveness have been reported. Nevertheless, we previously showed that human immunodeficiency virus (HIV)-infected subjects who did not respond to a hepatitis B virus (HBV) vaccine had a higher baseline frequency of Tregs than those who did respond [24, 31]. The fact that high baseline Treg frequencies impaired the response to vaccination in the elderly and HIV-infected subjects reinforces Treg increases in the steady states of both scenarios as a common feature of immunosenescence [27], which could compromise the ability of the immune system to mount a proper immune response [21–26, 31]. Further research about the underlying mechanism of these immunosenescence-related Treg increases is needed to develop novel approaches aimed to improve vaccine responsiveness in these scenarios.

In addition, we observed higher frequencies of Treg subsets expressing the proliferation marker Ki67 in non-responders. Heightened Treg proliferation has been shown in subjects exposed to infectious agents, in individuals with systemic autoimmunity and within tumours [32]. Along the same line, proliferating Tregs are associated with hyperactivation and disease progression in chronic HIV infection [33]. Furthermore, we found higher frequencies of all the CD4 and CD8 maturational subsets expressing the proliferation marker Ki67 in the non-responders to the influenza vaccine than the responders. In a recent work, we observed an inverse association between the magnitude of the HBV vaccine response and the frequency of proliferating CD4 T-cells in a cohort of HIV-infected patients [31]. As far as we know, no other previous evidence has associated a poor response to vaccination with conventional T-cell proliferation. However, Stervbo et al. reported an age-dependent association between influenza vaccine responsiveness and the proliferation of γδ T-cells [34].

The inflammatory status has been consistently shown to disturb vaccine responsiveness in the elderly [12, 13]. Moreover, hsCRP levels predict herpes zoster vaccine responses in elderly nursing home residents [35]. Although we failed to observe higher hsCRP levels in the non-responders, we observed a tendency towards higher levels of other inflammation-related markers such as D-dimers, neutrophils or the NLR in the non-responders. The fact that the anti-CMV titre was not associated with the response to the vaccine deserves a special mention, since CMV seropositivity has been previously associated with a negative effect on influenza vaccine responses [36, 37]. Nevertheless, as expected, we observed an association between the anti-CMV titre and several inflammation-related biomarkers (the ESR and LBP levels).

Interestingly, the limited CD4 T-cell repertoire diversity in aged individuals, probably a consequence of reduced thymic function, has been associated with a poor response to influenza vaccination in a mouse model [38]. However, in our cohort, we failed to observe lower thymic function in the non-responders compared with the responders. Nevertheless, we observed a higher frequency of ki67+ naïve T-cells, which is a surrogate marker of T-cell activation and proliferation, in the non-responders. Along this line, Sauce et al. [16] showed an association between increased naive T-cell turnover and decreased thymic function in elderly subjects, young adults thymectomised during early childhood and HIV-infected subjects. Importantly, despite 30% of our cohort showing thymic failure, this cohort could globally have a partially preserved thymic function, which can be appreciated when comparing the thymic function of our cohort with that of a different elderly population with a similar age range and thymic function values quantified by the same technique [39]. Alternatively, the higher Ki67 expression in the T-cell subsets of the non-responders could better reflect their inflammatory status, since we report consistent associations between Ki67 expression and several soluble inflammation-related parameters. Along this line, previous data link the inflammatory environment of HIV infection with increased memory CD4 T-cell cycling [40]. Thus, reasonably, this age-dependent homeostatic dysregulation involving T-cell proliferation could contribute to inflammaging. Interestingly, we also observed negative associations between thymic function and the levels of different inflammation-related biomarkers (mainly D-dimers but also the ESR, PLR and hsCRP levels), and a relationship between thymic involution and chronic systemic inflammation has also been previously described [41]. Thus, one can speculate that in the ageing context, thymic function and inflammation could be inversely interrelated. Accordingly, again in comparison with the cohort of Ferrando-Martínez et al. [39], our cohort shows a trend towards a lower inflammatory status while showing higher levels of thymic function.

Our study has several limitations. First of all, this is an exploratory and descriptive analysis with a relatively small size and our results need to be corroborated in higher cohorts. However, it supports and extends previous observations from aging studies in other human T-cell subsets and our rough observations raise interesting new questions in the immunosenescence topic. Additionally, recording deaths during a year of follow-up was not an objective of this study, and we got a low number of events and did not consider potential confounders for the levels of the biomarkers assessed, about possible concomitant anti-inflammatory treatments (such as statins or aspirin) for example. However, it is worth mentioning that the six subjects who died during the follow-up year showed lower thymic function but higher proliferation in the T-cell subsets, including the Treg subsets, as well as higher levels of inflammation-related biomarkers than those who survived during this follow-up period. In this sense, hsCRP levels have been previously associated with time to death in the elderly, and the risk of death is further elevated when high hsCRP levels are present in addition to CMV seropositivity [42] or low thymic function, as we previously reported [39]. Although we observed a higher disability degree in those subjects who died during the follow-up, our findings suggest that both thymic function and age-dependent homeostatic dysregulation involving T-cell proliferation (probably as a compensatory mechanism) could be relevant to the underlying mechanisms of progression to death in elderly people, and larger studies are encouraged to corroborate this hypothesis.

## Conclusions

In summary, age-dependent homeostatic dysregulation involving the proliferation of CD4 and CD8 T-cell subsets, including Tregs, seem related to a reduced responsiveness to influenza vaccination as well as to a higher inflammatory status in an elderly population. Our data support and extend previous observations from ageing studies of other human T-cell subsets and suggest that further research on the mechanisms underlying such relationships in the elderly could help to find better strategies to produce a proper vaccine response against influenza in this compromised population. Deepening this knowledge will also be useful to further understand how immunosenescence limits immune capacities.

## Methods

### Study design

We included elderly subjects from the Heliopolis Nursing Home, Seville, who were going to be vaccinated against influenza virus during November 2015 (the 2015–2016 campaign). Among these subjects, those older than 60 years, without cognitive impairment and able to sign the informed consent were included in this study. Subjects treated with antitumour therapy or any treatment that could influence their immune status (mainly corticosteroids) during the preceding 6 months were excluded. The vaccination protocol (Additional file 7: Figure S2) consisted of one intradermal dose of the trivalent influenza vaccine for the Northern Hemisphere (Intanza 15 μg, Sanofi Pasteur MSD, Lyon, France) with split and inactivated viruses of the strains: A/California/7/2009 H1N1pdm09, A/Switzerland/9715293/2013 H3N2 and B/Phuket/30731/2013 Yamagata lineage. Blood samples were collected pre-vaccination (from 29 to 0 days before the administration of the vaccine) and post-vaccination (from 12 to 33 days after vaccination) and processed at the Institute of Biomedicine of Seville, Virgen del Rocío University Hospital. We recorded the comorbid medical conditions for all the nursing home residents included in this study (details in Additional file 5: Table S5), as well as disability degree by the Barthel index for Activities of Dayly Living (ADL); in this score, 100 is totally independent whereas < 20 is totally dependent. Deaths occurring within one year after vaccination were recorded, except for one subject who was lost to follow-up due to a residency change. The study was approved by the Ethics Committee of the Virgen del Rocío University Hospital.

### Haemagglutination inhibition (HAI) test and vaccine responsiveness

Influenza vaccine responses were measured at the Microbiology Service of the Virgen de las Nieves University Hospital, Granada through an HAI test analysis. Pre-vaccination and post-vaccination sera were tested for HAI titres. The standardized antigen for the HAI test was prepared using the 2015–2016 trivalent influenza vaccine for the Northern Hemisphere (Influvac, Mylan Pharmaceuticals, Barcelona, Spain). The standardized antigen contained 4 haemagglutinin (HA) units per 25 μl of each of the following inactivated strains: A/California/7/2009 H1N1pdm09, A/Switzerland/9715293/2013 H3N2 and B/Phuket/30731/2013 Yamagata lineage. The HAI tests were performed with chicken red blood cells (RBCs) according to the WHO standard procedures [30]. Briefly, serum samples were pre-treated with Receptor Destroying Enzyme (RDE II Seiken, Denka Seiken Co Ltd., Tokyo, Japan) in order to inactivate non-specific haemagglutination inhibitors according to the manufacturer’s instructions. The RDE-treated sera were diluted 1:10 and then 25 μl was diluted 2-fold in PBS and incubated at room temperature for 15 min with 25 μl of standardized antigen. Then, 50 μL of standardized RBCs were added to each well and incubated for 30 min at room temperature. The HAI titre was the last dilution at which haemagglutination was inhibited. Seroprotection was defined as an HAI titre ≥40. A positive response was defined as a 4-fold or greater increase in the HAI titre between the pre- and post-vaccination serum samples [43]. For the aim of our study, we tested as a whole the response to all three vaccine strains and we defined a positive response having responded to at-least-one of the vaccine strains and a negative response having no response to any of the vaccine strains. That way we could discriminate those immune systems lacking the ability to mount a full immune response to the vaccination stimuli and those with the capability of productively react to such stimuli.

### Flow cytometry

Peripheral blood mononuclear cells (PBMCs) collected pre-vaccination were isolated from fresh blood and cryopreserved until analysis. The characterization of peripheral CD4 and CD8 T-cells was performed according to the distribution of their maturational subsets [naïve (CD27^**+**^CD45RA^**+**^), central memory (CD27^**+**^CD45RA^**−**^), effector memory (CD27^**−**^CD45RA^**−**^), terminally differentiated effector memory (TemRA) (CD27^**−**^CD45RA^**+**^) and recent thymic emigrants (RTEs; naïve CD31^**+**^)]. Our gating strategy is shown in Additional file 8: Figure S3. We measured the expression of an activation marker (HLA-DR), a senescence marker (CD57), an apoptosis susceptibility marker (CD95), a proliferation marker (Ki67) and a suppression marker (CTLA-4). Representative FACS plots for Ki67 staining are shown in Additional file 9: Figure S4. We also identified total-Tregs (CD25^hi^FoxP3^+^), naïve-Tregs (nTregs, CD45RA^+^FoxP3^lo^), effector-Tregs (eTregs, CD45RA^−^FoxP3^hi^) and non-Tregs (CD45RA^−^FoxP3^lo^) as previously described by Miyara et al. [44]. We studied the expression of the abovementioned activation, proliferation and suppression markers and a functional marker (CD39) on these Treg subsets.

For immunophenotyping, PBMCs were thawed and stained with the following surface antibodies: anti-CD31 PE-CF594, anti-CD56 BV510, anti-CD25 BV605, anti-CD45RA BV650, anti-CD4 BV786, anti-CD3 APC-H7 (BD Biosciences, USA), anti-CD39 FITC, anti-CD57 PE-Cy7, anti-HLA-DR BV570, anti-CD95 BV711 and anti-CD27 AF700 (BioLegend, USA). For intracellular staining, the cells were then fixed and permeabilized according to the manufacturer’s instructions (FoxP3/Transcription Factor Staining Buffer, eBioscience, USA) and intracellularly stained [anti-Ki67 PerCP-Cy5.5, anti-FoxP3 PE and anti-CTLA4 APC antibodies (BD Biosciences, USA)]. In each experiment, isotype controls for the antibodies specific for CD39, CD31, CD25, CD95, Ki67, FoxP3 and CTLA4 were included. The identification of viable cells was performed using LIVE/DEAD fixable Aqua Blue Dead Cell Stain (Life Technologies, USA). One million cells from each sample were stained, and a minimum of 100,000 total lymphocyte events were acquired. Flow cytometry was performed on an LSR Fortessa (BD Biosciences, USA). Analyses were performed using FlowJo version 9.3 (TreeStar).

### Sj/β-TREC ratio quantification

Thymic function was determined with pre-vaccination PBMC DNA by quantifying the sj/β-TREC ratio with a technique previously optimized by our group [43], with minor modifications. A schematic representation of the sj/β-TREC ratio quantification protocol is shown in the original report [45]. Briefly, in the same PCR reaction tube, the six dβJβ-TREC from cluster one were amplified, whereas the sj-TREC was amplified in a different PCR reaction tube. Twenty amplification rounds were performed to guarantee an accurate quantification at the real-time PCR step. All amplicons, dβJβ and sj-TREC, were then amplified together in a second round of PCR using the LightCycler® 480 System (Roche, Mannheim, Germany). We defined an sj/β-TREC ratio value lower than 10 as thymic function failure, since we previously found that this cutoff could forecast survival in a cohort of elderly people [39] as well as other clinical endpoints such as cytomegalovirus disease after solid organ transplantation [46] or HIV disease progression [47].

### Laboratory measurements and assaying soluble biomarkers

All determinations were performed with pre-vaccination samples. Absolute numbers of CD4^+^ and CD8^+^ T-cells and percentages of lymphocytes, monocytes, neutrophils, basophils, eosinophils and platelets were determined with an Epics XL-MCL flow cytometer (Beckman-Coulter, Brea, California). The high sensitivity C-reactive protein (hsCRP) and β2-microglobulin levels were determined with an immunoturbidimetric sera assay using Cobas 701 (Roche Diagnostics, Mannheim, Germany). The D-dimer levels were measured with an automated latex enhanced immunoassay using plasma samples (HemosIL D-Dimer HS 500, Instrumentation Laboratory, Bedford, Massachusetts). The mean corpuscular volume (MCV), mean platelet volume (MPV) and erythrocyte sedimentation rate (ESR) were determined with a Sysmex XN-200 analyser (Sysmex, Kobe, Japan). The platelet to lymphocytes ratio (PLR) and neutrophil to lymphocyte ratio (NLR) were calculated as inflammatory indices.

Serum and plasma samples were aliquoted and stored at − 20 °C until subsequent analysis of the levels of Interleukin-6 (IL-6), soluble CD163 (sCD163), and Lipopolysaccharide Binding Protein (LBP) as well as anti-CMV IgG antibody titres by colorimetric enzyme-linked immunosorbent assays (ELISA) according to manufacturer’s instructions. Specifically, the following kits were used: IL-6 (Quantikine® HS ELISA, R&D Systems, Minneapolis, Minnesota), sCD163 (MacroCD163™, IQProducts, Groningen, The Netherlands), LBP (Human ELISA kit, Hycult Biotech, Uden, The Netherlands), and anti-CMV IgG (Cytomegalovirus IgG ELISA Kit, Abnova, Taiwan, China).

### Statistical analysis

Continuous variables were recorded as medians and interquartile ranges [IQR], and categorical variables were recorded as the number of cases and percentages. Comparisons among groups were made using the nonparametric Mann–Whitney *U*-test for continuous variables and the χ2 or Fisher exact test for categorical variables. Correlations were assessed using Spearman’s rho correlation coefficient. A *p* value < 0.05 was considered statistically significant. Corrections for multiple comparisons were performed when indicated, by the Bonferroni Test or the Benjamini-Hochberg procedure, although both of them yielded similar results. Statistical analyses were performed using SPSS software (version 22; IBM SPSS, Chicago, USA), and graphs were generated using Prism (version 5, GraphPad Software, Inc.).

## Additional files

Additional file 1: **Table S1.** Characterization of Treg subsets in relation to the response to the influenza vaccine. (DOCX 16 kb)

Additional file 2: **Table S2.** Comparison of CD4 and CD8 T-cell subsets in groups defined by the vaccine response to the influenza vaccine. (DOCX 16 kb)

Additional file 3: **Table S3.** Associations among different inflammation-related and haematological parameters and the expression of Ki67 in T-cell subsets. (DOCX 21 kb)

Additional file 4: **Table S4.** Inflammation-related biomarkers and Ki67 expression in the T-cells of the subjects who died during the follow-up-year. (DOCX 18 kb)

Additional file 5: **Table S5.** Comorbid medical conditions recorded for the study. (DOCX 14 kb)

Additional file 6: **Figure S1.** Baseline and post-vaccination HAI titres. Data from the Haemagglutination Inhibition (HAI) test, which was performed at baseline (circles) and post-vaccination (squares), are shown as data for the whole population (*n* = 60) and the groups of influenza vaccine non-responders (*n* = 33) and responders (*n* = 27). Median [IQR] values are included in the data cells below each case. HAI titres were measured as a whole as the response to the three vaccine strains as indicated in the method section. (TIF 234 kb)

Additional file 7: **Figure S2.** Protocol. Subjects were vaccinated with one intradermal dose of the trivalent influenza vaccine Intanza (15 μg). Blood samples were collected pre-vaccination (from 29 to 0 days before the administration of the vaccine) and post-vaccination (from 12 to 33 days after vaccination). HAI titres were measured in the pre-vaccination and post-vaccination samples. T-cell immunophenotypes and soluble biomarkers were measured in the pre-vaccination samples. Deaths occurring within one year after vaccination were recorded. (TIF 123 kb)

Additional file 8: **Figure S3.** Gating strategy for the T-cell subsets. The gating strategy for the different CD4 T-cell subsets (naïve, central memory, effector memory and TemRA) depending on their expression of CD27 and CD45RA is represented. (TIF 894 kb)

Additional file 9: **Figure S4.** Representative FACS plots of ki67 staining. Treg subsets (naïve and effector Treg) and the non-Treg subsets were gated on CD4 T-cells depending on their expression of CD45RA and FoxP3. Then, the percentage of Ki67+ T-cells from each subset was quantified by using isotype control as it is shown in representative histograms. (TIF 455 kb)

## Data Availability

The corresponding author has taken custody of all data generated during this work. The FlowJo files generated during flow cytometry experiments as well as the clinical analytics and the database generated for statistical analyses are available upon request, respecting individual data protection.

## Abbreviations

APC: Antigen Presenting Cells
CM: Central Memory
CMV: Cytomegalovirus
ELISA: Enzyme-Linked Immunosorbent Assays
EM: Effector Memory
ESR: Erythrocyte Sedimentation Rate
eTreg: Effector Treg
HA: Haemagglutinin
HAI: Haemagglutination Inhibition
HBV: Hepatitis B Virus
HIV: Human Immunodeficiency Virus
hsCRP: High Sensitivity C Reactive Protein
IL-6: Interleukin-6
IQR: Interquartile Ranges
LBP: Lipopolysaccharide Binding Protein
MCV: Mean Corpuscular Volume
MPV: Mean Platelet Volume
NLR: Neutrophil to Lymphocyte Ratio
nTreg: Naïve Treg
PBMCs: Peripheral Blood Mononuclear Cells
PLR: Platelet to Lymphocyte Ratio
RBCs: Red Blood Cells
RDE: Receptor Destroying Enzyme
RTEs: Recent Thymic Emigrants
sCD163: Soluble CD163
sj/β-TREC: Signal Joint/β T-Cell Receptor Excision Circles
TemRA: Terminally Differentiated Effector Memory
Treg: Regulatory T-cell
WHO: World Health Organization
