# Supplementary material for: Effect of homeostatic T-cell proliferation in the vaccine responsiveness against influenza in elderly people
Source: Immun Ageing. 2019 Jul 5;16:14. doi: 10.1186/s12979-019-0154-y (PMC6612162; doi:10.1186/s12979-019-0154-y)
Supplement: Supplementary file 1 — Table S1. Characterization of Treg subsets in relation to the response to the influenza vaccine. (DOCX 16 kb) [file 12979_2019_154_MOESM1_ESM.docx]

**Table S1. Characterization of Treg subsets in relation to the response to the influenza vaccine.**

| **Parameter** | **Non-Responders**  **N=33** | **Responders**  **N=27** | ***p*** |
| --- | --- | --- | --- |
| **total-Treg** | 1.49 [1.08-1.85]­ | 1.12 [0.94-1.63] | *0.061* |
| **HLADR^+^** | 12.30 [7.51-17.60] | 11.30 [9.48-14.50] | 0.888 |
| **Ki67^+^** | 16.91 [11.58-19.95] | 14.75 [11.55-16.88] | 0.204 |
| **CTLA4^+^** | 60.56 [49.41-69.89] | 56.14 [49.29-60.96] | 0.262 |
| **CD39^+^** | 76.81 [43.54-85.85] | 65.90 [45.46-79.59] | 0.547 |
| **nTreg** | 6.42 [4.31-8.57] | 5.54 [3.55-8.30] | 0.336 |
| **HLADR^+^** | 0.80 [0.48-1.11] | 0.87 [0.47-1.04] | 0.795 |
| **Ki67^+^** | 38.90 [19.20-42.85] | 19.50 [16.10-35.60] | **0.025*** |
| **CTLA4^+^** | 6.57 [5.85-8.00] | 6.57 [6.06-7.92] | 0.797 |
| **CD39^+^** | 27.90 [12.60-42.35] | 35.30 [12.90-42.20] | 0.733 |
| **eTreg** | 2.86 [1.84-4.33] | 2.87 [2.12-4.16] | 0.873 |
| **HLADR^+^** | 9.270 [4.470-14.60] | 6.81 [4.59-11.30] | 0.705 |
| **Ki67^+^** | 10.90 [6.46-19.00] | 10.00 [7.52-12.90] | 0.683 |
| **CTLA4^+^** | 48.20 [33.65-56.25] | 37.00 [31.20-48.20] | 0.152 |
| **CD39^+^** | 48.00 [31.40-61.90] | 43.00 [24.60-60.70] | 0.499 |
| **nonTreg** | 20.29 [14.84-26.52] | 21.18 [17.83-30.95] | 0.353 |
| **HLADR^+^** | 2.06 [1.19-3.56] | 1.59 [1.35-3.24] | 0.688 |
| **Ki67^+^** | 38.50 [24.95-44.10] | 27.70 [17.90-40.40] | *0.053* |
| **CTLA4^+^** | 10.60 [7.65-15.70] | 9.20 [7.93-12.30] | 0.365 |
| **CD39^+^** | 33.20 [21.85-39.85] | 24.00 [17.00-42.90] | 0.305 |

Percentage of cells expressing each marker among the indicated subset. Continuous variables are expressed as median values [IQR]. Comparisons between the groups were made using the nonparametric Mann–Whitney *U* test. Variables with a *p* value <0.1 are shown in *italics*. Variables with a *p* value <0.05 were considered statistically significant and are shown in bold. *After the Bonferroni correction for multiple comparisons this comparison did not remain as statistically significant. Note: nTreg, naïve-Treg; and eTreg, effector-Treg.
