# Supplementary material for: Effect of homeostatic T-cell proliferation in the vaccine responsiveness against influenza in elderly people
Source: Immun Ageing. 2019 Jul 5;16:14. doi: 10.1186/s12979-019-0154-y (PMC6612162; doi:10.1186/s12979-019-0154-y)
Supplement: Supplementary file 2 — Table S2. Comparison of CD4 and CD8 T-cell subsets in groups defined by the vaccine response to the influenza vaccine. (DOCX 16 kb) [file 12979_2019_154_MOESM2_ESM.docx]

**Table S2. Comparison of CD4 and CD8 T-cell subsets in groups defined by the vaccine response to the influenza vaccine.**

| **Parameter** | **Non-Responders**  **N=33** | **Responders**  **N=27** | ***p*** |
| --- | --- | --- | --- |
| **CD4** |  |  |  |
| **CD4 RTE** | 52.20 [39.95-58.65] | 53.00 [43.30-66.00] | 0.199 |
| **CD4 naïve** | 28.50 [18.45-43.75] | 22.70 [14.40-43.60] | 0.385 |
| **CD4 CM** | 32.60 [22.25-41.35] | 34.00 [29.50-45.20] | 0.256 |
| **CD4 EM** | 32.00 [16.75-39.20] | 27.70 [18.90-43.10] | 0.761 |
| **CD4 TemRA** | 2.59 [1.45-5.83] | 1.94 [1.00-4.85] | 0.418 |
| **CD4 HLADR^+^** | 1.24 [0.76-2.52] | 1.19 [0.74-2.18] | 0.923 |
| **CD4 Ki67^+^** | 2.30 [1.85-2.80] | 2.02 [1.56-2.68] | 0.237 |
| **CD4 CD57^+^** | 10.49 [5.88-21.22] | 9.67 [4.27-18.04] | 0.377 |
| **CD4 CD95^+^** | 66.20 [49.15-80.50] | 70.80 [53.40-86.20] | 0.645 |
| **CD4 CTLA4^+^** | 8.035 [6.38-10.68] | 7.98 [6.90-9.58] | 0.700 |
| **CD8** |  |  |  |
| **CD8 RTE** | 96.20 [93.85-97.85] | 95.50 [90.00-98.70] | 0.824 |
| **CD8 naïve** | 6.985 [4.80-11.40] | 6.08 [3.50-8.82] | 0.216 |
| **CD8 CM** | 15.70 [9.455-23.15] | 19.20 [12.90-27.30] | 0.373 |
| **CD8 EM** | 25.60 [18.65-36.05] | 24.30 [17.40-35.30] | 0.778 |
| **CD8 TemRA** | 33.90 [26.75-55.55] | 35.30 [23.20-52.90] | 0.677 |
| **CD8 HLADR^+^** | 2.53 [1.47-4.07] | 3.14 [1.76-4.21] | 0.325 |
| **CD8 Ki67^+^** | 9.48 [7.94-12.05] | 9.66 [6.55-12.35] | 0.325 |
| **CD8 CD57^+^** | 55.00 [40.50-70.10] | 52.70 [39.80-66.00] | 0.744 |
| **CD8 CD95^+^** | 97.80 [96.35-98.30] | 97.90 [96.18-98.83] | 0.609 |

Frequencies of the maturational subsets and the percentage of cells expressing each marker among the CD4 and CD8 T-cell subsets. Continuous variables are expressed as median values [IQR]. Comparisons between the groups were made using the nonparametric Mann–Whitney *U* test. Variables with a *p* value <0.05 were considered statistically significant and are shown in bold. Note: RTE, recent thymic emigrants; CM, central memory; EM, effector memory; and TemRA, terminally differentiated effector memory.
