# Supplementary material for: Effect of homeostatic T-cell proliferation in the vaccine responsiveness against influenza in elderly people
Source: Immun Ageing. 2019 Jul 5;16:14. doi: 10.1186/s12979-019-0154-y (PMC6612162; doi:10.1186/s12979-019-0154-y)
Supplement: Supplementary file 3 — Table S3. Associations among different inflammation-related and haematological parameters and the expression of Ki67 in T-cell subsets. (DOCX 21 kb) [file 12979_2019_154_MOESM3_ESM.docx]

**Table S3. Associations among different inflammation-related and haematological parameters and the expression of Ki67 in T-cell subsets.**

|  | **LBP**  **(ng/mL)** | **hsCRP**  **(mg/L)** | **B2M**  **(µg/mL)** | **DD**  **(µg/L)** | **% Lymph** | **% Mono** | **% Neutro** | **% Baso** | **% Eosino** | **Platelets**  **(x10e9/L)** | **MCV**  **(fL)** | **MPV**  **(fL)** | **PLR** | **NLR** |
| --- | --- | --- | --- | --- | --- | --- | --- | --- | --- | --- | --- | --- | --- | --- |
| **% total-Treg** |  |  |  | **0.329**  **0.013** |  |  |  | 0.235  0.071 |  |  | -0.241  0.064 | -0.221  0.089 |  |  |
| **% nTreg Ki67**^+^ |  |  |  | 0.254  0.059 | -0.217  0.095 |  |  | **0.430***  **0.001** |  |  | -0.223  0.087 | **-0.550***  **<0.001** |  |  |
| **% eTreg Ki67**^+^ |  | **0.301**  **0.020** |  | 0.254  0.059 |  |  |  | **0.269**  **0.038** |  |  |  | **-0.242**  **0.062** |  |  |
| **% nonTreg Ki67**^+^ |  |  |  |  | -0.227  0.081 |  |  | **0.401***  **0.001** | -0.243  0.063 |  |  | **-0.411***  **0.001** |  | 0.220  0.091 |
| **% CD4 Ki67**^+^ |  |  | **0.332**  **0.010** | **0.446***  **0.001** |  | **0.264**  **0.041** |  |  |  |  |  |  | **0.278**  **0.031** |  |
| **% CD4 Naïve Ki67**^+^ |  |  |  |  | **-0.266**  **0.040** |  |  | **0.494***  **<0.001** |  | **0.291**  **0.024** | -0.223  0.087 | **-0.510***  **<0.001** |  | 0.247  0.057 |
| **% CD4 CM Ki67**^+^ |  |  |  |  | -0.233  0.073 |  | 0.225  0.084 | **0.424***  **0.001** | -0.249  0.057 | 0.239  0.066 |  | **-0.415***  **0.001** |  | 0.225  0.083 |
| **% CD4 EM Ki67**^+^ |  |  |  |  | **-0.310**  **0.016** |  | **0.307**  **0.017** | **0.425***  **0.001** | -0.233  0.076 | 0.249  0.055 |  | **-0.470***  **<0.001** | 0.239  0.066 | **0.309**  **0.016** |
| **% CD4 TemRA Ki67**^+^ | **0.295**  **0.029** |  |  |  |  |  |  | **0.360**  **0.005** | -0.224  0.090 | 0.231  0.079 |  | **-0.469***  **<0.001** |  |  |
| **% CD8 Ki67**^+^ | **0.277**  **0.041** | -0.219  0.096 |  |  |  |  |  |  |  |  |  |  |  |  |
| **% CD8 Naïve Ki67**^+^ |  |  |  | **0.269**  **0.045** |  |  |  | **0.397***  **0.002** |  | 0.217  0.095 | **-0.297**  **0.021** | **-0.517***  **<0.001** | 0.242  0.062 |  |
| **% CD8 CM Ki67**^+^ |  |  |  |  |  |  |  | **0.406***  **0.001** |  | 0.235  0.071 | **-0.298**  **0.021** | **-0.531***  **<0.001** | 0.231  0.076 |  |
| **% CD8 EM Ki67**^+^ |  |  | 0.224  0.088 |  |  |  |  | **0.408***  **0.001** |  | **0.267**  **0.039** | **-0.327**  **0.011** | **-0.551***  **<0.001** | 0.217  0.096 |  |
| **% CD8 TemRA Ki67**^+^ |  |  |  |  |  |  |  | **0.434***  **0.001** |  | 0.247  0.057 | **-0.303**  **0.019** | **-0.550***  **<0.001** |  |  |

Correlations were assessed using Spearman's rho correlation coefficient. Variables with a *p* value <0.1 are shown in *italics*. Variables with a *p* value <0.05 were considered statistically significant and are shown in bold. *After the Bonferroni correction for multiple comparisons, those correlations remaining statistically significant are highlighted. N=60. Note: LBP, Lipopolysaccharide Binding Protein; hsCRP, high sensitivity C-reactive protein; B2M, β2-microglobulin; DD, D-Dimers; Lymph, lymphocytes; Mono, monocytes; Neutro, neutrophils; Baso, basophils; Eosino, eosinophils; MCV, mean corpuscular volume; MPV, mean platelet volume; PLR, platelet to lymphocyte ratio; NLR, neutrophils to lymphocyte ratio; nTreg, naïve-Treg; eTreg, effector-Treg; CM, central memory; EM, effector memory; and TemRA, terminally differentiated effector memory.
