# Supplementary material for: Effect of homeostatic T-cell proliferation in the vaccine responsiveness against influenza in elderly people
Source: Immun Ageing. 2019 Jul 5;16:14. doi: 10.1186/s12979-019-0154-y (PMC6612162; doi:10.1186/s12979-019-0154-y)
Supplement: Supplementary file 4 — Table S4. Inflammation-related biomarkers and Ki67 expression in the T-cells of the subjects who died during the follow-up-year. (DOCX 18 kb) [file 12979_2019_154_MOESM4_ESM.docx]

**Table S4. Inflammation-related biomarkers and Ki67 expression in the T-cells of the subjects who died during the follow-up-year.**

|  | **Death (YES)**  **N=6** | **Death (NO)**  **N=53** | ***P**** |
| --- | --- | --- | --- |
| **sj/β TREC ratio** | 0 [0-0] | 34 [10-51] | **0.001** |
| **Thymic failure ^(#)^** | 6 (100) | 13 (25) | **<0.001** |
| **hsCRP (mg/L)** | 5.10 [3.70-7.58] | 2.60 [1.25-4.30] | **0.012** |
| **PLR** | 152 [152-229] | 116 [87-166] | 0.318 |
| **NLR** | 2.75 [2.30-3.57] | 2.12 [1.63-2.85] | 0.153 |
| **β2-microglobulin (µg/mL)** | 3.50 [2.45-5.40] | 2.50 [2.03-3.30] | *0.076* |
| **% nTreg Ki67^+^** | 49.65 [34.78-51.15] | 29.60 [16.70-39.65] | **0.007** |
| **% eTreg Ki67^+^** | 18.75 [11.49-23.23] | 9.96 [6.50-13.95] | **0.034** |
| **% nonTreg Ki67^+^** | 45.05 [34.93-51.60] | 36.10 [20.65-41.20] | **0.025** |
| **% CD4 Ki67^+^** | 2.65 [2.28-3.20] | 2.08 [1.72-2.69] | *0.096* |
| **% CD4 N Ki67^+^** | 32.65 [20.39-42.28] | 21.70 [12.00-30.35] | 0.160 |
| **% CD4 CM Ki67^+^** | 39.30 [29.70-48.78] | 28.20 [19.15-36.90] | *0.068* |
| **% CD4 EM Ki67^+^** | 39.30 [30.80-44.05] | 28.50 [19.75-35.60] | **0.041** |
| **% CD4 TemRA Ki67^+^** | 39.60 [30.93-42.73] | 25.50 [18.75-33.30] | **0.033** |
| **% CD8 Ki67^+^** | 7.60 [4.20-12.35] | 9.86 [7.79-12.18] | 0.242 |
| **% CD8 N Ki67^+^** | 47.55 [32.12-55.28] | 15.20 [8.52-42.45] | **0.039** |
| **% CD8 CM Ki67^+^** | 38.15 [26.11-49.18] | 17.70 [14.45-34.10] | *0.057* |
| **% CD8 EM Ki67^+^** | 42.15 [30.74-48.70] | 20.40 [15.75-36.60] | **0.020** |
| **% CD8 TemRA Ki67^+^** | 41.05 [24.07-41.95] | 18.70 [12.30-32.55] | **0.032** |
| **Comorbidities (number^1^)** | 4.5 [2.7-6] | 3 [2-5] | 0.211 |
| **Barthel index^2^** | 65 [51-76] | 90 [72-100] | **0.013** |
| **< 20** | 0 | 3 (5.7) |  |
| **20-35** | 0 | 2 (3.7) |  |
| **40-55** | 2 (33.3) | 3 (5.7) |  |
| **≥ 60** | 4 (66.6) | 28 (52.8) |  |
| **100** | 0 | 17 (32.1) |  |

Comparisons between the groups were made using the nonparametric Mann–Whitney *U* test. Variables with a *p* value <0.1 are shown in *italics*. Variables with a *p* value <0.05 were considered statistically significant and are shown in bold. *After correction for multiple comparisons by the Benjamini-Hochberg procedure, applying a 10% FDR, all statistical significances remained. Note: hsCRP, high sensitivity C-Reactive Protein; PLR, platelets to lymphocyte ratio; NLR, neutrophils to lymphocyte ratio; nTreg, naïve-Treg; eTreg, effector-Treg; N, naïve; CM, central memory; EM, effector memory; and TemRA, terminally differentiated effector memory. **^(#)^** Thymic failure is defined as an sj/β TREC ratio<10. ^1^Details of comorbidities recorded are shown in Table S5. ^2^100 is totally independent and <20 is totally dependent.
