# Supplementary material for: Effect of homeostatic T-cell proliferation in the vaccine responsiveness against influenza in elderly people
Source: Immun Ageing. 2019 Jul 5;16:14. doi: 10.1186/s12979-019-0154-y (PMC6612162; doi:10.1186/s12979-019-0154-y)
Supplement: Supplementary file 5 — Table S5. Comorbid medical conditions recorded for the study. (DOCX 14 kb) [file 12979_2019_154_MOESM5_ESM.docx]

**Table S5. Comorbid medical conditions recorded for the study.**

| Cardiovascular diseases | Hypertension, Ischemic cardiopathy, Chronic venous insufficiency, Stroke, Atrial fibrillation, Arteriopathy |
| --- | --- |
| Metabolic disorders | Type-II Diabetes, Hypothyroidism, Hypercholesterolemia, Obesity |
| Bone/Joint diseases | Bone fracture, Osteoporosis, Arthrosis, Arthritis |
| Brain disorders | Psichiatric disorders, dementia, Parkinson disease, Acoustic neurinoma |
| Respiratory diseases | EPOC |
| Cancer | Prostate carcinoma, Lung carcinoma, Bladder cancer, |
| Digestive diseases | Hiatal hernia, Diverticulitis, Colon polyposis, Alcoholic liver disease, Hepatic steatosis |
| Genitourinary pathology | Kidney failure, Prostatic hyperplasia |
| Dermatological diseases | Psoriasis |
| Habits/Addictions | Smoking habit, Alcoholism |
