# Supplementary figures and images for: Effect of homeostatic T-cell proliferation in the vaccine responsiveness against influenza in elderly people
Source: Immun Ageing. 2019 Jul 5;16:14. doi: 10.1186/s12979-019-0154-y (PMC6612162; doi:10.1186/s12979-019-0154-y)

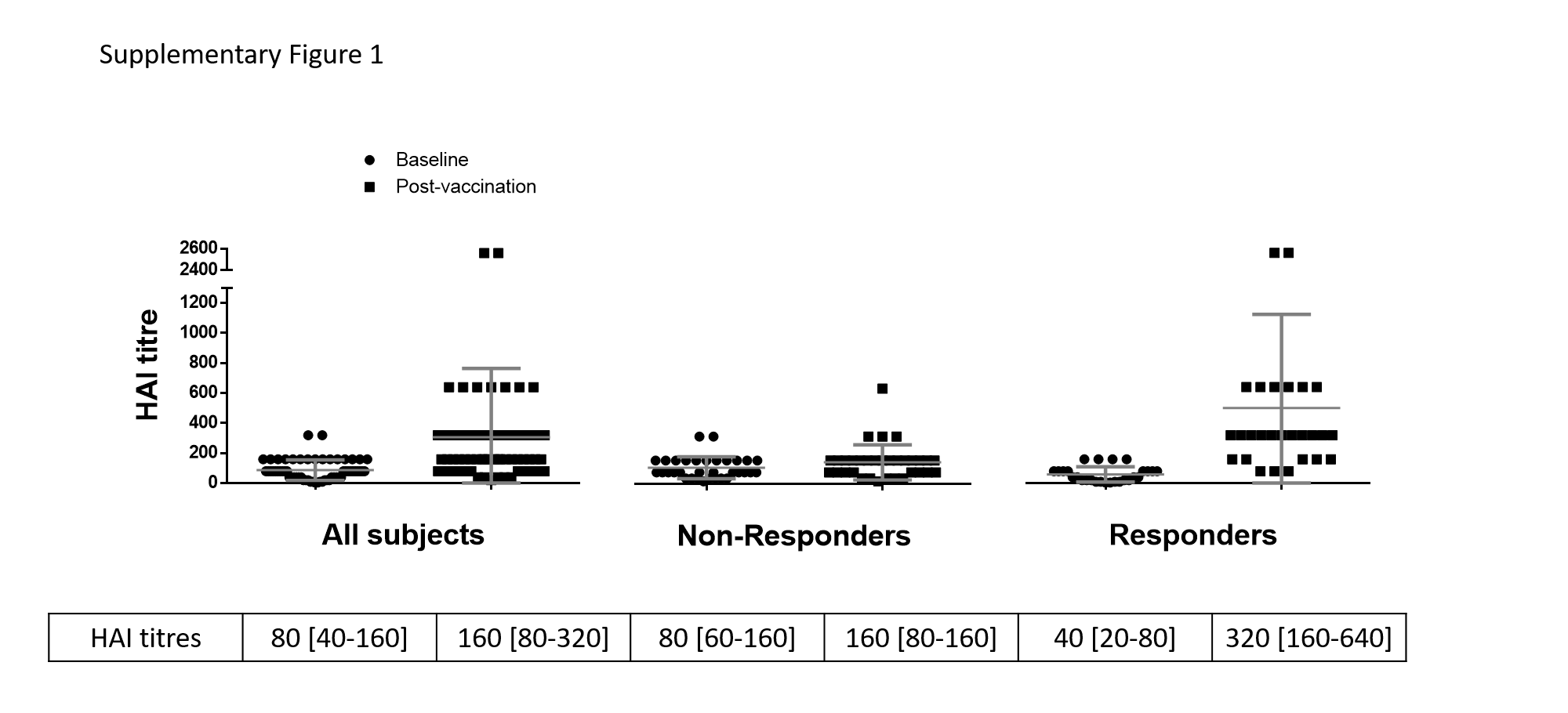

Supplement: Supplementary file 6 — Figure S1. Baseline and post-vaccination HAI titres. Data from the Haemagglutination Inhibition (HAI) test, which was performed at baseline (circles) and post-vaccination (squares), are shown as data for the whole population (n = 60) and the groups of influenza vaccine non-responders (n = 33) and responders (n = 27). Median [IQR] values are included in the data cells below each case. HAI titres were measured as a whole as the response to the three vaccine strains as indicated in the method section. (TIF 234 kb) [file 12979_2019_154_MOESM6_ESM.tif]

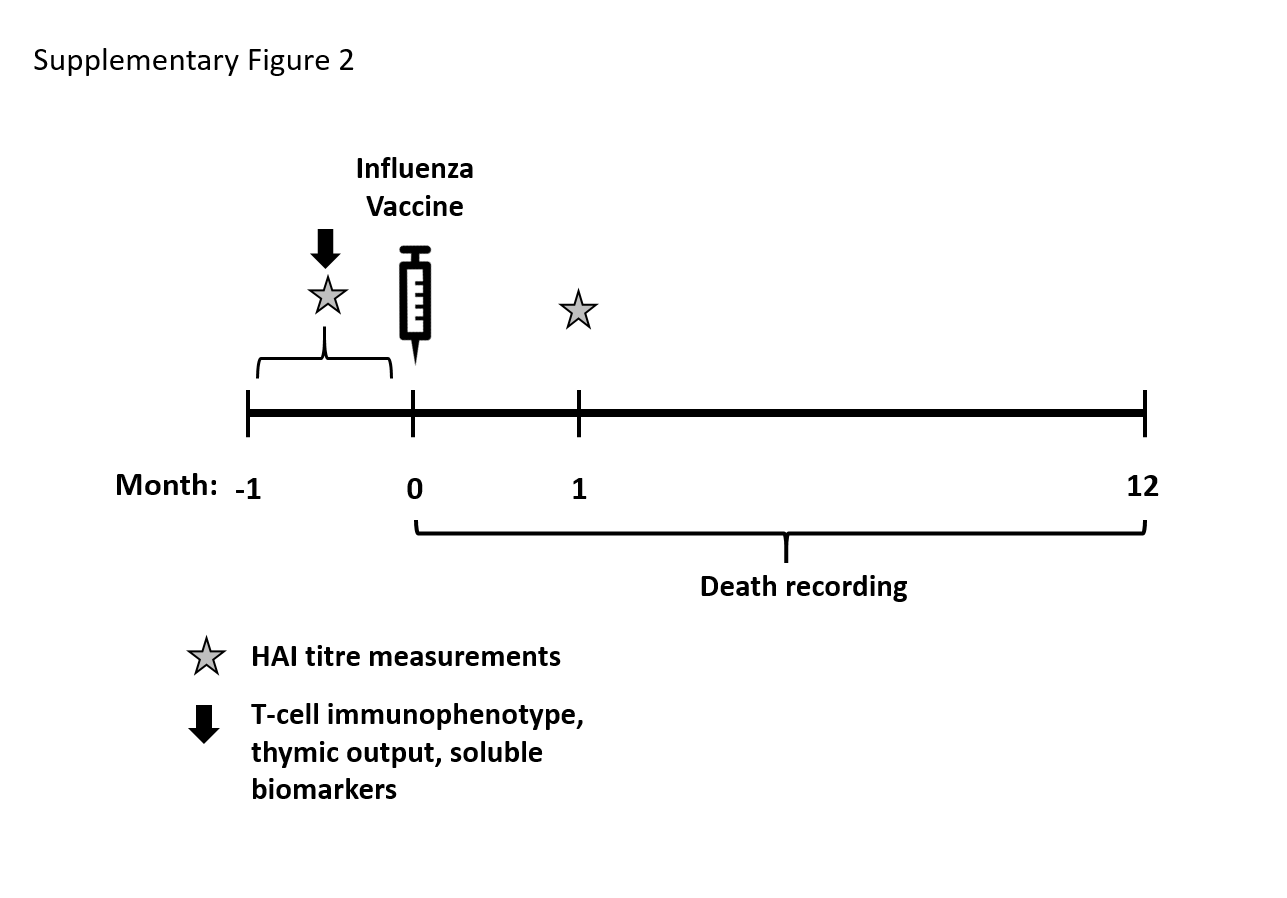

Supplement: Supplementary file 7 — Figure S2. Protocol. Subjects were vaccinated with one intradermal dose of the trivalent influenza vaccine Intanza (15 μg). Blood samples were collected pre-vaccination (from 29 to 0 days before the administration of the vaccine) and post-vaccination (from 12 to 33 days after vaccination). HAI titres were measured in the pre-vaccination and post-vaccination samples. T-cell immunophenotypes and soluble biomarkers were measured in the pre-vaccination samples. Deaths occurring within one year after vaccination were recorded. (TIF 123 kb) [file 12979_2019_154_MOESM7_ESM.tif]

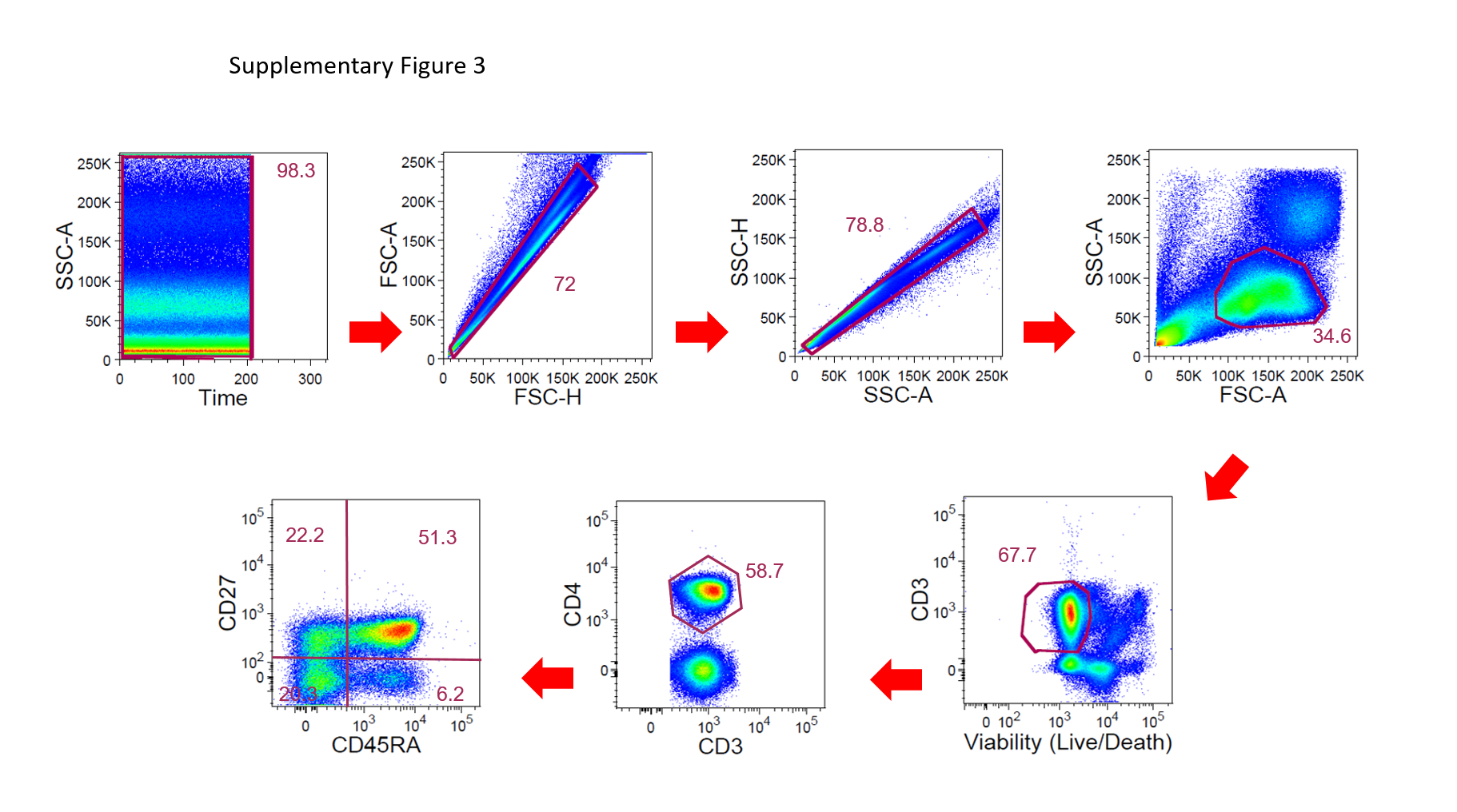

Supplement: Supplementary file 8 — Figure S3. Gating strategy for the T-cell subsets. The gating strategy for the different CD4 T-cell subsets (naïve, central memory, effector memory and TemRA) depending on their expression of CD27 and CD45RA is represented. (TIF 894 kb) [file 12979_2019_154_MOESM8_ESM.tif]

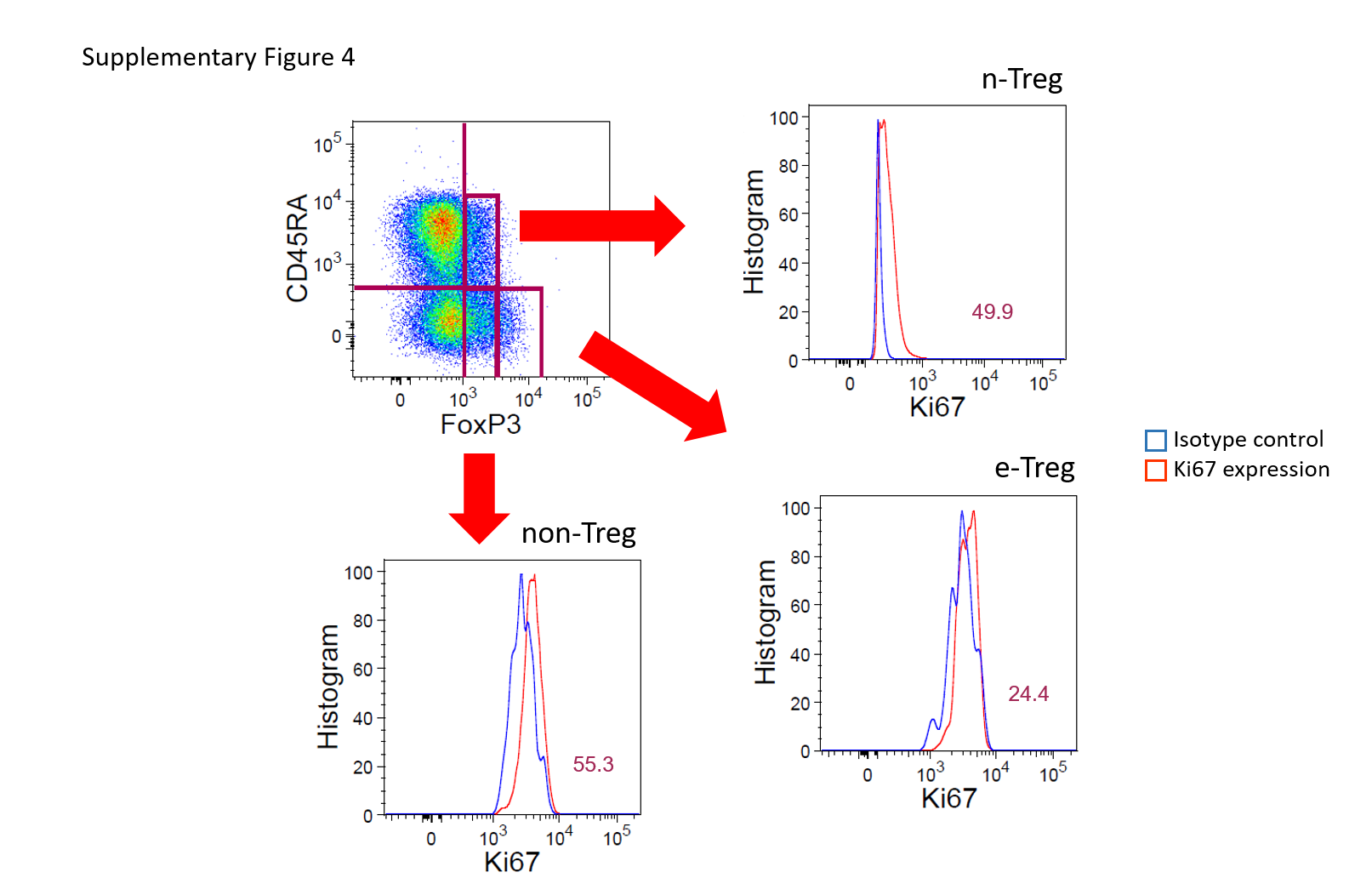

Supplement: Supplementary file 9 — Figure S4. Representative FACS plots of ki67 staining. Treg subsets (naïve and effector Treg) and the non-Treg subsets were gated on CD4 T-cells depending on their expression of CD45RA and FoxP3. Then, the percentage of Ki67+ T-cells from each subset was quantified by using isotype control as it is shown in representative histograms. (TIF 455 kb) [file 12979_2019_154_MOESM9_ESM.tif]
